# Thinking outside the Laboratory: Analyses of Antibody Structure and Dynamics within Different Solvent Environments in Molecular Dynamics (MD) Simulations

**DOI:** 10.3390/antib7030021

**Published:** 2018-06-24

**Authors:** Mohammed M. Al Qaraghuli, Karina Kubiak-Ossowska, Paul A. Mulheran

**Affiliations:** 1 Department of Chemical and Process Engineering, University of Strathclyde, Glasgow G1 1XJ, UK; karina.kubiak@strath.ac.uk (K.K.-O.); paul.mulheran@strath.ac.uk (P.A.M.); 2 Department of Physics, University of Strathclyde, Glasgow G4 0NG, UK

**Keywords:** implicit solvent, explicit solvent, root-mean-square distance (RMSD), root-mean-square fluctuations (RMSF), antibody, molecular dynamics (MD)

## Abstract

Monoclonal antibodies (mAbs) have revolutionized the biomedical field, directly influencing therapeutics and diagnostics in the biopharmaceutical industry, while continuing advances in computational efficiency have enabled molecular dynamics (MD) simulations to provide atomistic insight into the structure and function of mAbs. Despite the success of MD tools, further optimizations are still required to enhance the computational efficiency of complex mAb simulations. This issue can be tackled by changing the way the solvent system is modelled to reduce the number of atoms to be tracked but must be done without compromising the accuracy of the simulations. In this work, the structure of the IgG_2a_ antibody was analyzed in three solvent systems: explicit water and ions, implicit water and ions, and implicit water and explicit ions. Root-mean-square distance (RMSD), root-mean-square fluctuations (RMSF), and interchain angles were used to quantify structural changes. The explicit system provides the most atomistic detail but is ~6 times slower in its exploration of configurational space and required ~4 times more computational time on our supercomputer than the implicit simulations. Overall, the behavior of the implicit and explicit simulations is quantifiably similar, with the inclusion of explicit ions in the implicit simulation stabilizing the antibody to reproduce well the statistical fluctuations of the fully explicit system. Therefore, this approach holds promise to maximize the use of computational resources to explore antibody behavior.

## 1. Introduction

Monoclonal antibody (mAb)-based therapeutics represent a large and growing fraction of all drug candidates that have emerged to phase 3 clinical trials in 2015 [1]. This dominance is reflected in the annual number of antibody therapeutics granted a first approval, which has reached double-digits (total of 10) for the first time in 2017 [2]. The unprecedented interest in these “Y” shaped glycoproteins has originated from their capability to recognize various antigens or molecular targets with extremely high affinity. This has enabled the wide adaptation of these antibodies in both diagnostic and therapeutic applications. 

**Figure 1:**
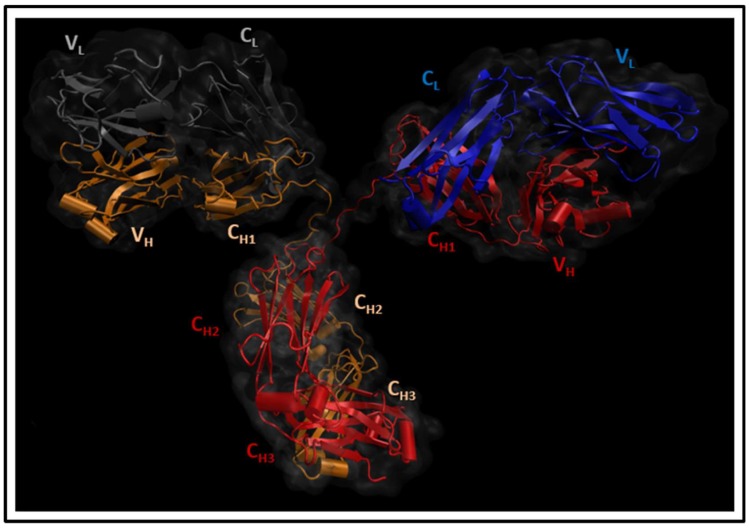
Antibody structure.

An antibody, as illustrated in Figure 1, generally comprises two heavy and two light polypeptide chains [3]. Any of the nine characterized heavy-chain subtypes can be linked to either a lambda (λ) or kappa (k) light chain to create one of the nine antibody subclasses in humans (IgM, IgD, IgG_1–4_, IgA_1–2_, or IgE) [4]. Since a large proportion of serum immunoglobulins is of the IgG type [5], and most of the therapeutic antibodies that are licensed or currently in development utilize an IgG backbone [6], this isotype is studied here.

IgG_2a_ antibody (PDB ID: 1IGT): Four chains (A, B, C, and D) were viewed by VMD [7] in cartoon format, where barrels, ribbon arrows, and loose strings indicate α-helices, β-sheets, and less structured parts regions (such as loops), respectively. The heavy chains were coloured as red (B) and orange (D), whilst the light chains were coloured as blue (A) and grey (C). The IgG antibody can be dissected into three fragments: two identical antigen-binding fragments (Fabs) that each contain the first two domains of the heavy (VH and CH1) and light (VL and CL) chains, and one crystallisable region fragment (Fc) consisting of two monomers, each comprising an N-glycosylated CH2 domain and a CH3 domain. 

Several tools and guidelines have been developed in both academic and commercial settings to enhance the characterization of antibodies, including structural and physicochemical properties, biological activity, selectivity and specificity, immunochemical properties, purity, impurities, and contaminants [8,9]. Despite the robustness of the developed approaches, the recent computational revolution has undoubtedly added an extra edge to the discovery and development of these bio-therapeutics. Computer-aided drug design (CADD) is being utilized to accelerate in silico hit identification, hit-to-lead selection, structural analysis, optimization of the pharmacokinetic/pharmacodynamics profile, and to anticipate binding and safety issues [10]. The diversified range of CADD applications has even spanned most stages of target identification, lead discovery, and expanded to preclinical or clinical trials [11,12]. 

Molecular dynamics (MD) simulations have been proposed as a predictive tool that can provide comprehensive atomistic insight into the complex structure of proteins and, in particular, antibodies. MD simulations have successfully provided information about interactions, flexibility, interfacial dynamics and electrostatics analysis, and binding of small and macrobiomolecules or DNA to nanoparticles [13,14,15]. Moreover, MD simulations were effectively used to analyze antibody–antigen interactions [16], antibody fragment conformational stability [17], thermal stress and deglycosylation impact on the antibody conformational stability [18], and to investigate the role of individual amino acids on the internal dynamics of antibodies [19]. Subsequently, MD simulations provide a prospective analysis that can guide a specific and site-directed optimization and engineering of new bio-therapeutics. 

Despite the aforementioned successes, MD simulations place significant limitations on the time and length scales that can be investigated. In general, MD simulations are performed by considering the solvent molecules explicitly, computing all interactions between the solute and solvent atoms. The fully explicit system can be considered as the most accurate and detailed approach for biomolecular simulations. However, the inclusion of explicit solvent molecules substantially reduces the computational efficiency [20]. Since computational cost and access to supercomputers can limit the uptake of these simulations, many strategies to facilitate efficient sampling of the protein configurational space have been developed, including coarse-grained modelling [21], adaptive biased dynamics [22], and implicit solvation [23]. Implicit solvation can accelerate simulations by approximating the discrete solvent as a continuum using the generalized Born (GB) model [23]. Such an approach considerably decreases the number of particles tracked in the system and leads to faster sampling of the conformational space, which is mainly due to the reduced effective viscosity of the solvent introduced by this approximate method [24]. Conformational sampling in implicit solvent simulations can be, for instance, ~100 times faster compared to common explicit solvent simulations [25,26].

The accuracy of the implicit-solvent approximations, explicit water models, and the interatomic force fields has been extensively discussed elsewhere [11,27,28]. However, the effect of two various solvent environments, explicit versus implicit systems, on the antibody structure and dynamics has not been studied hitherto. More understanding will undoubtedly guide the biopharmaceutical industry and antibody-based academic field to select the best simulation options based on the bespoke requirements of the biomolecules as well as computational resources. Consequently, the focus of this article will be to analyze the effect of different solvent and ionic environments on the structure of the analyzed IgG antibody. We have developed a clear understanding of how to analyze different proteins, like bovine serum albumin and lysozyme [29,30], and our aim here is to translate this knowledge into the antibody field. 

## 2. Materials and Methods

### 2.1. Molecular Dynamics Simulations

The simulations were performed with the NAMD 2.11 package [31], using the CHARMM27 force-field, and the results were analyzed using VMD 1.9.1 software [7]. Three sets of simulations were performed: (i) using explicit water and ions (“explicit” trajectories); (ii) using implicit water and ions (“implicit”); and (iii) implicit water but explicit ions (“explicit_ions”). The antibody studied is the IgG_2a_ mouse antibody, and the crystal structure was retrieved from the Protein Data Bank (PDB ID: 1IGT) [32].

**Figure 2:**
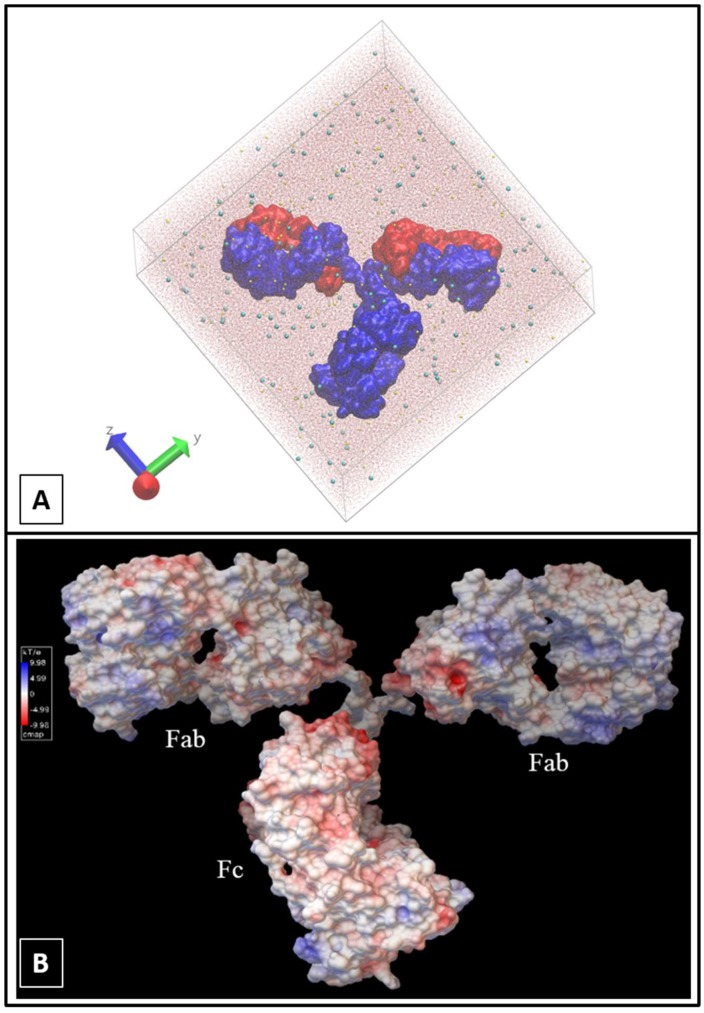
Antibody electrostatic potential and representation in primary simulation cell. (**A**) The explicit solvent (and ions) model illustrated by VMD [7]. The antibody is shown as a surface model; heavy chains are indicated in blue, while light chains are in red. Ions are shown by spheres: Cl^−^ in yellow and Na^+^ in cyan. The box is filled by water shown as transparent lines for clarity; (**B**) The surface-mapped electrostatic energy of IgG_2a_ was calculated and viewed by Python Molecule Viewer (PMV) v. 1.5.6 [34]. The map colour was coded as white: 0 kT/e, Blue: 9.98 kT/e, Red: −9.98 kT/e.

In the explicit simulation, the system was initially solvated in a cubic water box (TIP3P model) that extended at least 30 Å from any antibody atom. Then, NaCl salt at ionic strength I = 5 × 10^−2^ M was added to better reproduce experimental conditions (161 Na^+^ and 161 Cl^−^ ions were used in this case). The simulation system shown in Figure 2A was composed of 537,646 atoms in total (antibody: 20,148; water: 517,176; ions: 322), and the size of the obtained simulation box was 147 Å × 197 Å × 196 Å. The simulation was conducted through three stages: (i) water and ion minimization (1000 steps) and subsequent 100 ps run (integration step of 1 fs) in constant temperature of 300 K (trajectory D0); (ii) minimization of the entire system (10,000 steps) followed by 60 ps heating to 300 K and equilibration (integration step of 2 fs) at constant temperature for 5.94 ns (trajectory D1); and (iii) the production trajectory (D2) of 100 ns duration with a 2 fs time step. The antibody atoms were immobilized only during the initial minimization stage. The simulation was performed in the NVT ensemble with a Langevin thermostat as implemented in NAMD [31]. The cutoff for VdW interactions was 12 Å, while the PME method was used for electrostatics [33]. In total, we had six independent trajectories, each under different conditions, that showed similar general behavior, and here, analyzed in detail, is one with the single antibody in its water box. 

Water, as a dielectric medium, screens (or reduces) the electrostatic interactions between charged species, therefore, in the implicit method, it is modelled as a dielectric continuum. The GB approximation models ions as charged spheres, the internal dielectric constant of which is lower than that of the solvent. The screening that each charged atom experiences is, therefore, determined by its local environment; the more an atom is surrounded by other atoms, the less its electrostatic field is screened by the solvent. The GB method reduces the computational costs of the simulations, since fewer atomic positions are explicitly computed on each time step, although the computations are more involved using the GB electrostatic model. Hence, less computational time is required to calculate a given trajectory length and, simultaneously, the conformational space sampling is accelerated, while the speedup factor strongly depends on the system studied [24]. This makes the GB method very attractive when long-time simulations are required for relatively large systems. On the other hand, the quality of these trajectories needs to be examined carefully, since the lowered barriers in the implicit solvent models might lead and trap the system in irrelevant energy basins.

The implicit-water simulation protocol was kept as close to the above as possible. The GB implicit solvent model, as implemented in NAMD [31], was used with α cutoff 12 Å and ion concentration 5 × 10^−2^ M, modifying the default settings of the water medium. In all stages, the electrostatic and vdW cutoff was 14 Å as recommended in NAMD [31]. It is worth noting that methods using periodic boundary conditions cannot be combined with this implicit solvent model. The “implicit” production trajectory was 100 ns duration. 

It is instructive to view the electrostatic potential of the antibody surface in the GB implicit model, since this will help the interpretation of how explicit ions interact with it. The electrostatic potential of the selected IgG_2a_ antibody was calculated using Adaptive Poisson-Boltzmann Solver (APBS) Version 0.5.1 in the Python Molecule Viewer (PMV) Version 1.5.6 [34] (Compute > Electrostatics > Compute Potential using APBS). The produced energy was mapped to the surface with medium surface quality and at a 1 Å distance (Compute > Electrostatics > Map Potential to Surface) and this reproduces well the electrostatic potential of the protein within the GB model. The electrostatic potential map this produces is shown in Figure 2B.

The third “explicit_ions” simulation used implicit water solvent while explicitly including Na^+^ and Cl^−^ ions. Therefore, the ion concentration parameter was set to zero to not double-count the ions. Na^+^ and Cl^−^ ions were added at the same locations as found in the explicit simulations. The other simulation conditions and parameters were as in the previous “implicit” system. We note that, due to the lack of periodicity in the system, the explicit ions in the implicit solvent model are free to diffuse away from the protein. Nevertheless, careful initial positioning of the explicit ions (namely, close to positions the ions would obtain in the explicit simulation) prevents this; the particular charge distribution on the protein surface is an important factor influencing the explicit ion behavior. 

### 2.2. RMSD, RMSF, and Interchain Angles 

Both root-mean-square distance (RMSD) and fluctuations (RMSF) analyses were conducted using homemade Tcl scripts executed through the Tk Console of VMD [7]. RMSD is a widely used tool in bioinformatics that quantifies the variability of similar proteins, such as alternative conformations of the same protein [35]. As used here, this tool measures the degree of the similarity between two protein three-dimensional (3D) structures with the same number of atoms [36]. 

The RMSD is defined as

(1) $$\;RMSD=\sqrt{\frac{\sum_{i=1}^{N_{atoms}}{\left|{\vec{r}}_{i}\left(t_{1}\right)-{\vec{r}}_{i}\left(t_{2}\right)\right|}^{2}}{N_{atoms}}}$$

where *N_atoms_* is the number of atoms in the protein structure and ${\vec{r}}_{i}\left(t\right)$ is the position of the *i*^th^ atom at a given time *t*. In practice, to calculate the RMSD, the two protein structures to be compared are initially treated as rigid bodies (no internal flexibility allowed), then overlapped (aligned) using only translations and rotations. 

Another useful tool to track structural changes in proteins is RMSF, where the RMSD is calculated for each protein residue. It is frequently referred to as “fluctuations” because it reflects each residue’s mobility during the MD trajectory. RMSF reports an amplitude of residue movement (fluctuation) from the average position (in the aligned structures) over the entire length of the MD trajectory. Time average fluctuations of atoms belonging to the same residue were calculated from the formula

(2) $$\;RMSF_{k}=\sqrt{{\langle \frac{\sum_{i=1}^{N_{K}}{\left|{\vec{r}}_{i}\left(t\right)-{\langle {\vec{r}}_{i}\rangle }_{T}\right|}^{2}}{N_{K}}\rangle }_{T}}$$

where ${\vec{r}}_{i}\left(t\right)$ is the position of atom *i* in residue *k* at time *t*, *N_k_* is the number of atoms in the residue, and ${\langle {\vec{r}}_{i}\rangle }_{T}$ is the time average over the trajectory. Similar to RMSD, an extra component to the RMSF is introduced if two domains/chains change their relative orientations. To exclude these effects, we optimized our scripts to focus on each protein fragment. The most frequently used unit for RMSD and RMSF is Å (10^−10^ m), which is convenient for the protein length-scale. 

The large-scale movement of the Fab and Fc regions was monitored by selecting one amino acid at the end of these three regions (Fab-right: Lys 65, Fab-left: Asp 31, and Fc: Phe 442) and one amino acid from the central CH1–CH2 linker region (Cys 225). The angles between the three triplets (left angle: Asp31_Cys225_Phe442; right angle: Lys65_Cys225_Phe442; top angle: Asp31_Cys225_Lys65) throughout the simulations were measured using VMD [7].

Data underpinning this publication are openly available from the University of Strathclyde KnowledgeBase at http://dx.doi.org/10.15129/7e313c26-2064-4ad2-a143-ab874ae9877a.

## 3. Results and Discussion

**Figure 3:**
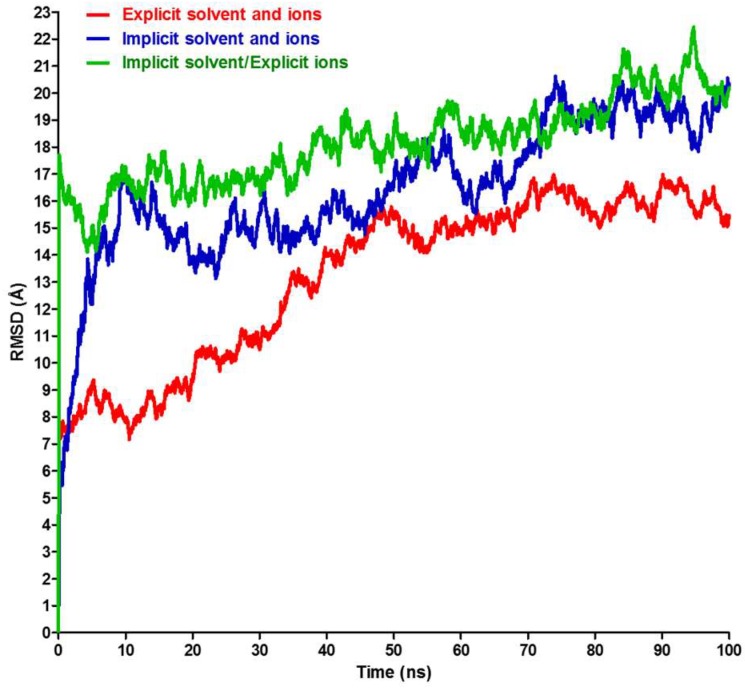
Total RMSD analysis of the entire antibody. The RMSD of the entire antibody (with all four chains included) for the three simulations described in the text.

General comparison of the total RMSD for the entire antibody in the three solvent environments reveal an overall stability of the antibody in these simulations (Figure 3). The explicit model shows lower RMSD values, especially during the first 50 ns (Supplementary Video S1) when compared to the two implicit models (with or without explicit ions, Supplementary Videos S2 and S3). The second 50 ns of the simulations show an overall tendency towards steady state behavior, fluctuating around 15–16 Å and 17–20 Å for the explicit and implicit simulations, respectively. The RMSD value has different significance and interpretation for long, medium, or short polypeptide chains because it depends on the number of atoms included in the structural alignment [35]. The RMSD values in Figure 3 can be considered reasonable for such a large, naturally flexible and complex protein and agrees with previously reported values for a similar antibody subclass [18] and with a human IgG_1_ monoclonal antibody (trastuzumab) [37].

**Figure 4:**
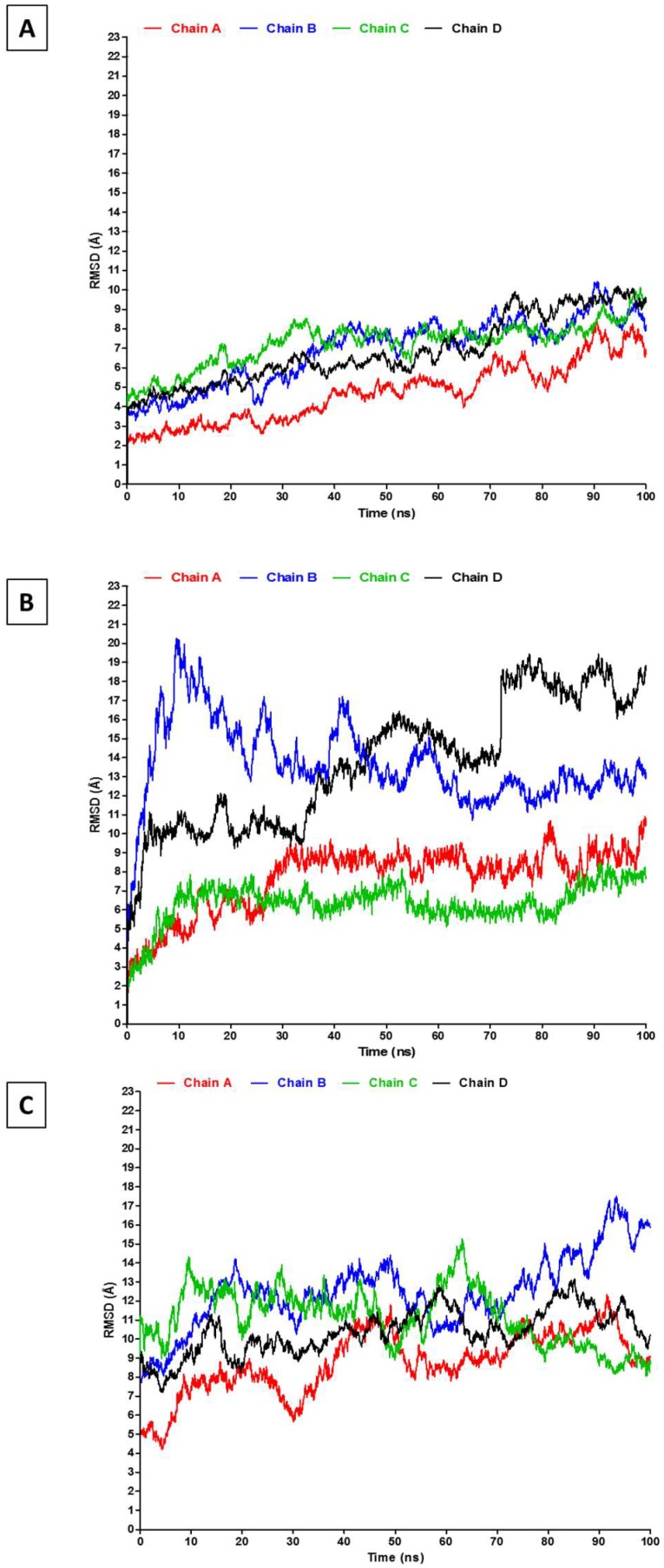
RMSD analysis of the four antibody chains. The RMSD of the two heavy chains (B and D) and light chains (A and C) for **A**: explicit; **B**: implicit; and **C**: explicit_ions simulations.

Despite this general agreement, it is necessary to understand the source of this difference in RMSD at different stages of each simulation and across the three simulations. Therefore, the RMSD analyses were extended to each of the four (two heavy and two light) chains of the antibody, as shown in Figure 4. The four chains show low and approximately similar RMSD in the explicit simulation (Figure 4A), with the lowest RMSD observed in one of the light chains (chain A). A similar RMSD trend is observed in the “explicit_ions” simulation, albeit at higher values (Figure 4C). For the “implicit” simulation, the two heavy chains (D and B) have interchangeably revealed very high RMSD when compared to the light chains (A and C) of the same simulation and to the other two simulations (Figure 4B). Therefore, the presence of explicit ions in the “explicit_ions” simulation appears to have stabilized the antibody throughout modification of its electrostatic environment.

As mentioned in the Materials and Methods, the implicit solvent approximation characterizes the solvent as a uniform dielectric medium instead of having explicit water molecules with internal charge distribution, polarity, and mobility. In practice, this means that no explicit interactions with water, including protein stabilization by H-bonds with water molecules or OH moieties or directly with ions, are possible. The applied ionic strength of 5 × 10^−2^ M homogenously modified the electrostatic properties of the medium containing the antibody so that the ionic sources of the field are not explicitly included. Contrary to that, in the “explicit_ions” system, all the sources of the electrostatic field (charged antibody residues as well as solvent ions) are explicitly present in the system, while the water is still represented as a homogenous medium. The explicit ions can now interact with the antibody in a similar way to the fully explicit simulation and this, in light of the presented RMSD analysis, seems to have a visible impact on maintaining the structural stability of the antibody, and this point will be discussed further below. 

**Figure 5:**
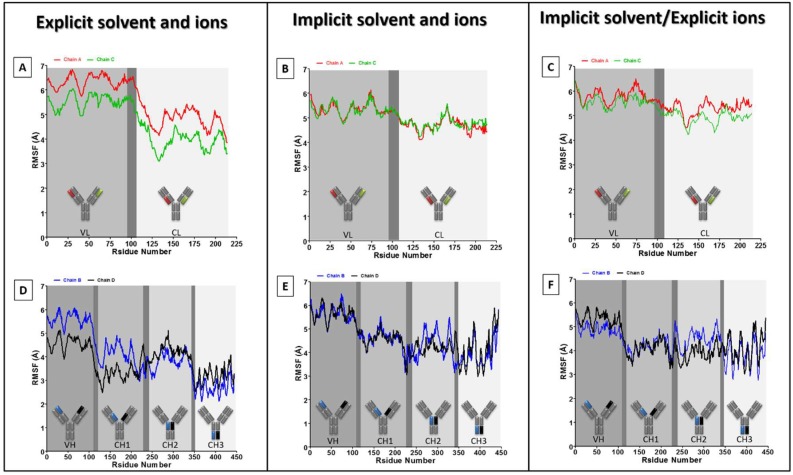
RMSF analysis of the four antibody chains. RMSF values plotted for each residue for the two light (**A**–**C**) and two heavy (**D**–**F**) chains. Each domain is depicted on the RMSF figures, separated by dark grey regions that represent the linkers. The inset schemes indicate the analyzed fragment location in the antibody 3D structure.

The RMSF of the four chains is shown in Figure 5, and these indicate the following. Firstly, as might be expected, amino acids within the variable domains (VH and VL) have generally fluctuated more than in the constant domains (CL, CH1, CH2, and CH3) (Figure 5A–F). This could be attributed to the tendency of antibodies to have flexible variable domains, which allow them to better search for and ultimately bind their target. Wang and colleagues have noticed a similar trend using deglycosylated IgG_2a_ antibody in an explicit system [18]. The same authors have additionally shown slight differences in RMSF between similar domains of the two identical heavy or light chains. Secondly, the main linker region (CH1–CH2 linker), which connects the two Fabs into the Fc region, did not show a very high RMSF, as was expected (Figure 5D–F). This is attributed to the fact that this CH1–CH2 linker is very short (10 amino acids) when compared to the regions they are connecting in the Fab (227 amino acids) and Fc (205 amino acids). Therefore, this linker would require small movement to implement large deviation in the overall Fab or Fc alignment (thus resembling door hinges). Thirdly, the CH3 domains of the “implicit” and “explicit_ions” simulations have fluctuated higher than the explicit simulation, especially towards the C-terminal of the antibody (Figure 5D–F). Furthermore, the “implicit” system video (Supplementary Video S2) demonstrates an unfolding of the C-terminal loops of the two heavy chains (B and D), which contributes to the high RMSF values of these CH3 domains (Figure 5E). Moreover, our previous experience that an RMSF of 5 Å for a residue in an α–helical structure is substantial and usually means that the helix structure is strongly affected or even unfolded, while the same value for a residue in a loop region or residue responsible for binding a ligand is modest. Thus, visual analysis of the structures is still necessary to interpret the RMSF values.

Visual analysis of the trajectory for the “explicit_ions” (some details are visible in Supplementary Move S3), indicates that the explicit Cl^−^ ions interact with the Fab C-terminus. The reason for this is apparent in Figure 2B, where we display the GB electrostatic potential on the antibody surface. The Fab regions tend to be positive and therefore attract the negative solute ions. These in turn provide a strong, local neutralization that is absent in the fully implicit solvent model, accounting for the stabilization we observe. This observation suggests that the explicit treatment of ions should be considered with the implicit water model in any protein system.

The RMSF analysis of the antibody structure indicates that the two light (A and C) and two heavy (B and D) chains are still composed of rigid structural domains (Light: CL and VL; heavy: VH, CH1, CH2, and CH3), as well as flexible linkers. To deconvolute the contribution of each to the overall structural changes, we calculated RMSD separately for each of the 12 domains, as presented in Supplementary Figures S1–S3. The CH1–CH2 linker region, the variable domains (VL and VH), and CH3 domains have relatively high RMSD values when compared to other domains and linker regions as shown on Supplementary Figures S1 and S2. Moreover, the RMSD values of the analyzed domains were lower in the explicit simulation than the two implicit simulations, reflecting a common tendency of the GB method to decrease the energy barriers and therefore speed-up conformational sampling [24]. This comprehensive RMSD analysis has mirrored the observed unfolding of the N- and C-terminals in the implicit simulations (Supplementary Figure S2), as additionally observed in the “implicit” video (Supplementary Video S2). The “explicit_ions” simulation has shown similar unfolding of the N-terminal VL (chain A), yielding the high RMSD values of Supplementary Figure S3A and Supplementary Video S3. However, this “explicit_ions” simulation was more stable than the “implicit” simulation, and the high RMSD values of CH3 domains (chains D and B) as well as VL (chains C), in Supplementary Figure S3B–D, did not reflect unfolding but an entire movement of the Fab region towards the Fc region. We emphasize that neither the domains, the Fab/Fc regions, nor the entire antibody unfold as indicated by the RMSD calculated for all the interesting fragments separately; the relatively large values obtained for longer fragments reflect the internal flexibility of the antibody. The corresponding RMSD plots are included in Supplementary Materials.

**Figure 6:**
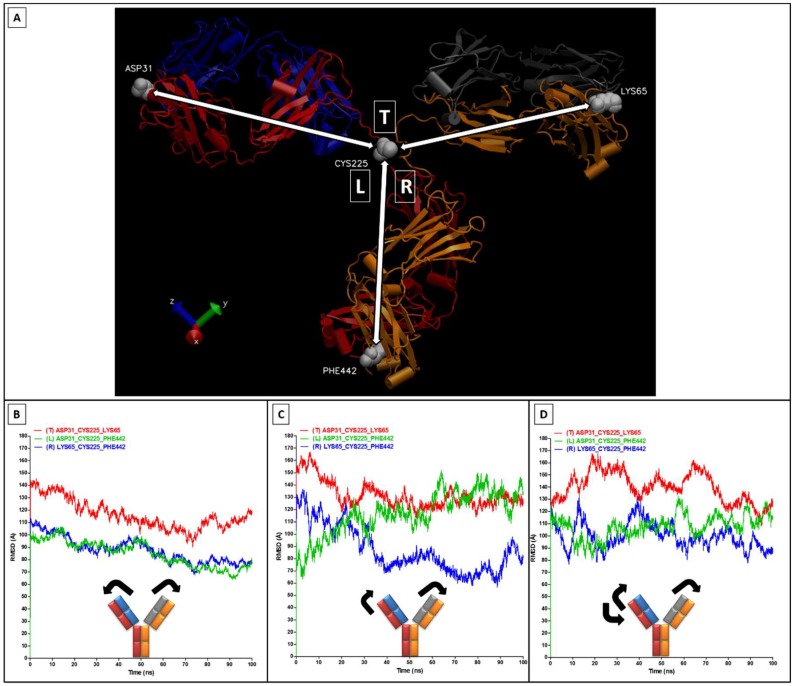
Movement analysis of the antibody Fabs and Fc fragments. Movements of the angles shown in (**A**); These angle changes were measured through each of the three simulations as illustrated in (**B**) explicit; (**C**) implicit; and (**D**) explicit_ions.

In order to better interpret the RMSD/RMSF values, it is necessary to measure the Fab/Fc movements by monitoring the angles across the CH1–CH2 linker (Figure 6A). The angles measured in the “implicit” simulation show movements of the right Fab towards the Fc region (reduction in angle R) combined by an opposite movement of the left Fab away from the Fc region (increase in angle L) (Figure 6C), and this contributed to the high RMSD values. In comparison, in the “explicit” simulation, the two Fab regions have slightly moved towards the Fc region (Figure 6B). The angles of the “explicit_ions” simulation have shown more general fluctuations of the Fabs (Figure 6D). It is important to note that movements of these Fc and Fab regions were not in a completely flat plane, and the sum of the angles is not consistently 360° (e.g., see Figure 6B).

**Figure 7:**
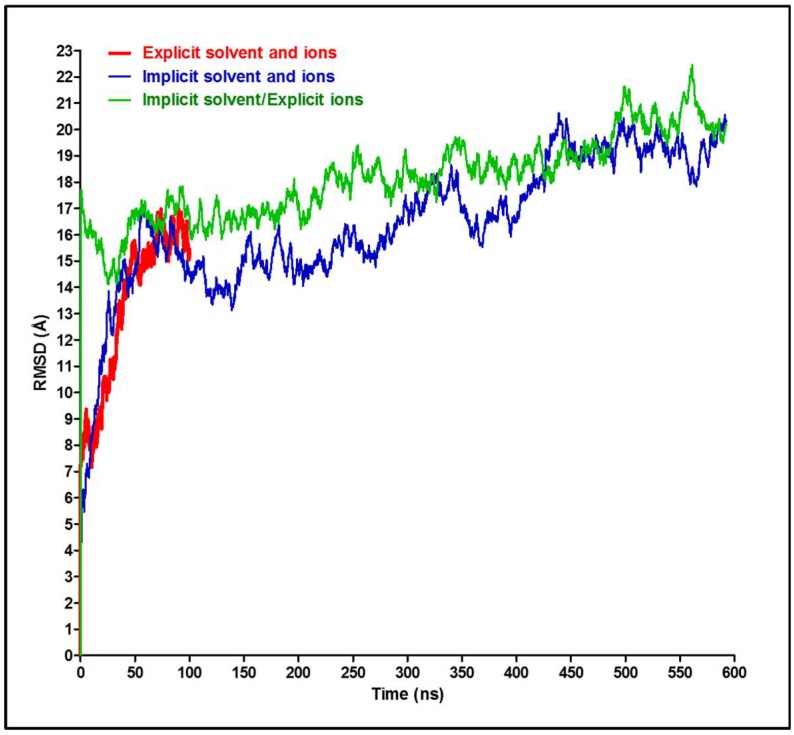
RMSD extrapolation between implicit and explicit systems. Time values were corrected for the implicit system through multiplying by a conversion factor of 6.

**Figure 8:**
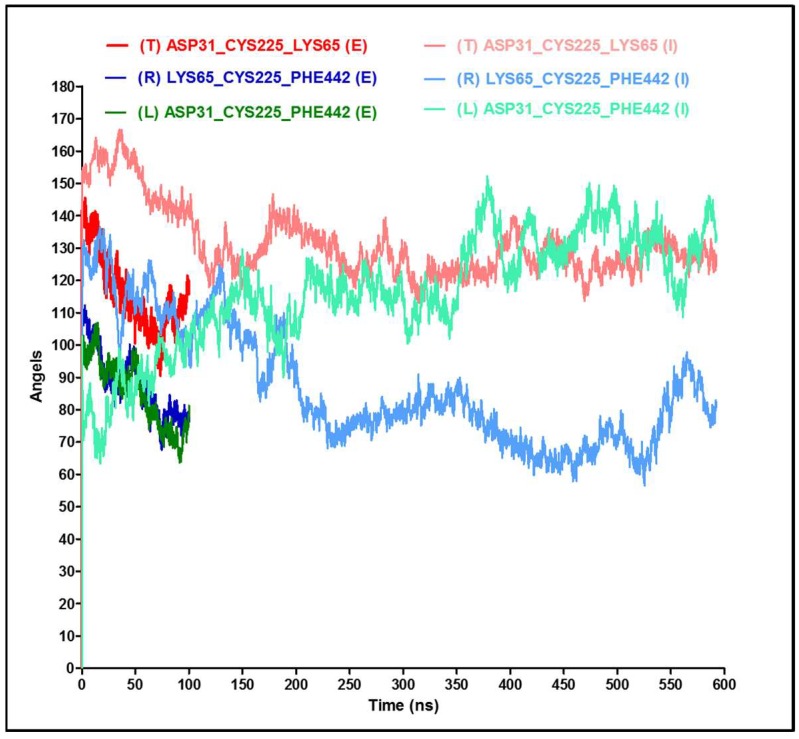
Angle extrapolation between implicit and explicit systems. Time values were corrected for the implicit system through multiplying by a conversion factor of 6.

Since it is understood that the implicit solvent simulation explores the conformational space faster than its explicit counterpart [24], it is interesting to verify this for the antibody model and determine the acceleration factor. Visual analysis of implicit trajectories revealed that the antibody achieves the most similar structure to the one observed after 100 ns of explicit trajectory after 16.88 ns of the simulation, which gives a speedup factor of ~6. Therefore, the elapsed time in the “implicit” simulation might be scaled by 6 when compared to the “explicit” time, as we have done for the RMSD plots shown in Figure 7. Interestingly, we see that the RMSD values of the “explicit” simulation have aligned well with the implicit models. Similar alignments were tested for the angles from Figure 5B,C; they show a similar trend for the top and right angles and for the periodicity of the left angel (Figure 8). Other researchers, based on the time required to achieve a particular conformation or on a defined angle flip frequency (depending on the system studied), have indicated the range of anticipated speedup of implicit systems from ~0.7-fold to ~60-fold for different structures, depending on the size of the molecules and the details of the transition [24]. The GB approximation in the implicit system is associated with poor algorithmic complexity that scales as ~n^2^ (n: number of solute atoms), whereas the PME-based explicit simulations scale as ~N logN (N: total number of solvent and solute atoms combined). Thus, even though n ≪ N in a typical simulation, the nominal computational speed of the GB model can become much lower than that of the corresponding explicit-solvent simulation for large systems (100,000 atoms or more) [26], whereas the speed of conformational sampling in implicit simulations can be ~2–20 times faster than explicit simulations [25,38]. Consequently, despite the algorithmic limitation of the GB model, the speedup due to conformational sampling can enhance the overall speedup and generate faster GB-based implicit models [24].

In our case, we find that the explicit simulations consumed 4 times more of the computational time (core-hours) on the ARCHIE-WeSt supercomputer than the implicit simulations. Therefore, the observed total speedup of the explicit method is around 24. 

## 4. Conclusions

Comparison of the antibody behavior in three different solvent environments were conducted to gain valuable structural analysis and to provide operational flexibility for antibody-based analysis. All three systems have shown an overall stability when the RMSD and RMSF of different sections of the antibody were analyzed. We estimate that the explicit system was ~6 times slower in exploring configurational space and consumed ~4 times more computing time (core hours) on our supercomputer when compared to implicit simulations. Therefore, the total speedup factor of the implicit solvent method in the case of IgG is estimated to be around 24. Although the quality (in the meaning of structural similarity to the initial conformation of the antibody) in the case of the explicit simulation was better than the implicit simulation, this could be attributed to the fact that the explicit simulation is only representing the first 16.88 ns of the implicit counterpart. In addition, the quality of the implicit simulation was enhanced by explicitly adding the ions to stabilize the electrostatic search within the system. This enhancement is evidenced by the loss of unfolding in the C-terminal loops when compared to the implicit simulation (as shown on Supplementary Videos S1–S3). Subsequently, the explicit_ions simulation can be used to predict the antibody behavior on the longer term and to perform a wider scan of possible conformations. On the other hand, the explicit model can be adopted to anticipate short-term behavior of this biomolecule with full atomistic detail. 

MD simulations represent a valuable tool to analyze the dynamics of movements in response to external factors such as solvent molecules and their targeted antigens. This allows a more accurate estimate of various applications, such as the thermodynamics and kinetics associated with antibody-target recognition, antibody mutation and humanization, CDRs grafting, homology modelling, binding to intrinsically disordered proteins, and antibody allostery. There is an ongoing argument to specify the accurate time length of these simulations to reflect the experimental environments. We have estimated a 100 ns of simulations time to be sufficient in this case. However, further runs of the explicit model for longer times will be always beneficial, if computational resources are available, and can be addressed in the future to allow further inspection of the hinge region. In addition, it will also be useful to analyze the effect of adding specific molecules at specific positions of the binding sites (both with implicit and explicit solvents) to examine their influence on the binding of these antibodies to their designated targets. Finally, this study can be extended to analyze other important antibody subclasses, like IgG1, and compare their structural differences.

## Supplementary Materials

The following are available online at http://www.mdpi.com/2073-4468/7/3/21/s1, Supplementary Figure S1: RMSD analysis of each chain (and component VL, CL, VH, CH1, CH2, CH3, and linker parts) in the explicit solvent and ions simulation, Supplementary Figure S2: RMSD analysis of each chain (and component parts) in the implicit solvent and ions simulation, Supplementary Figure S3: RMSD analysis of each chain (and component parts) in the implicit solvent/explicit ions simulation, Supplementary Video S1: explicit simulation, Supplementary Video S2: implicit simulation, and Supplementary Video S3: explicit_ions simulation.
