# Supplementary material for: Thinking outside the Laboratory: Analyses of Antibody Structure and Dynamics within Different Solvent Environments in Molecular Dynamics (MD) Simulations
Source: Antibodies (Basel). 2018 Jun 24;7(3):21. doi: 10.3390/antib7030021 (PMC6640683; doi:10.3390/antib7030021)
Supplement: Supplementary file 1 [file antibodies-07-00021-s001.zip › Supplementary files/Supplementary Figures.docx]

**Supplementary data**


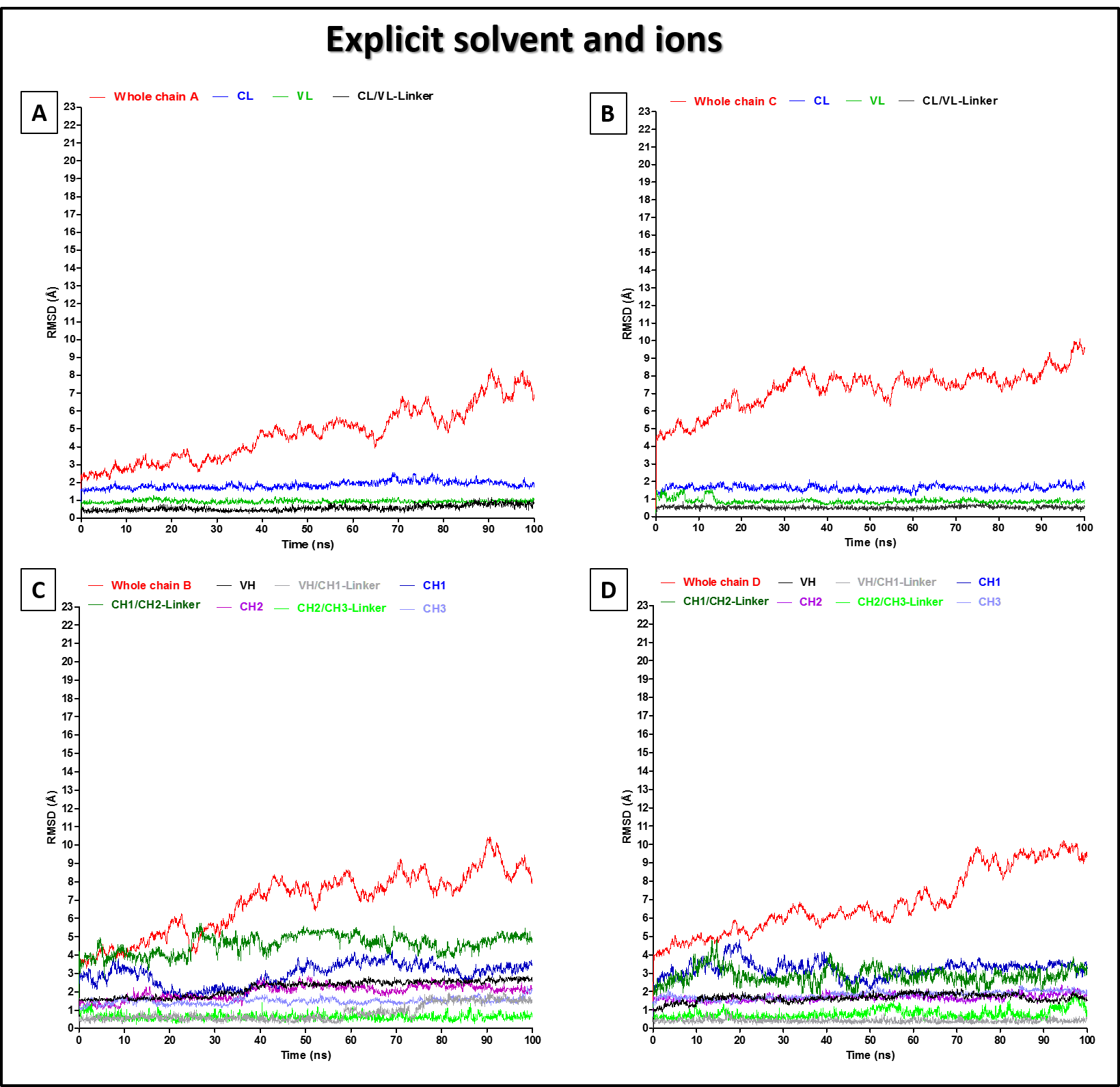


**Supplementary Figure S1: RMSD analysis of each chain in the explicit solvent and ions simulation**

The RMSD of various domains and linkers of the antibody four chains are illustrated in A) light chain A; B) light chain C; C) heavy chain B; D) heavy chain D. Each light chain is composed of variable (VL) and constant (CL) domains connected by a linker (CL/VL –linker). Whilst the heavy chain is composed of variable (VH) and three constant (CH1, CH2, and CH3) domains. Three linkers (VH/CH1-Linker, CH1/CH2-Linker, and CH2/CH3-Linker) connect the four domains of the heavy chains.


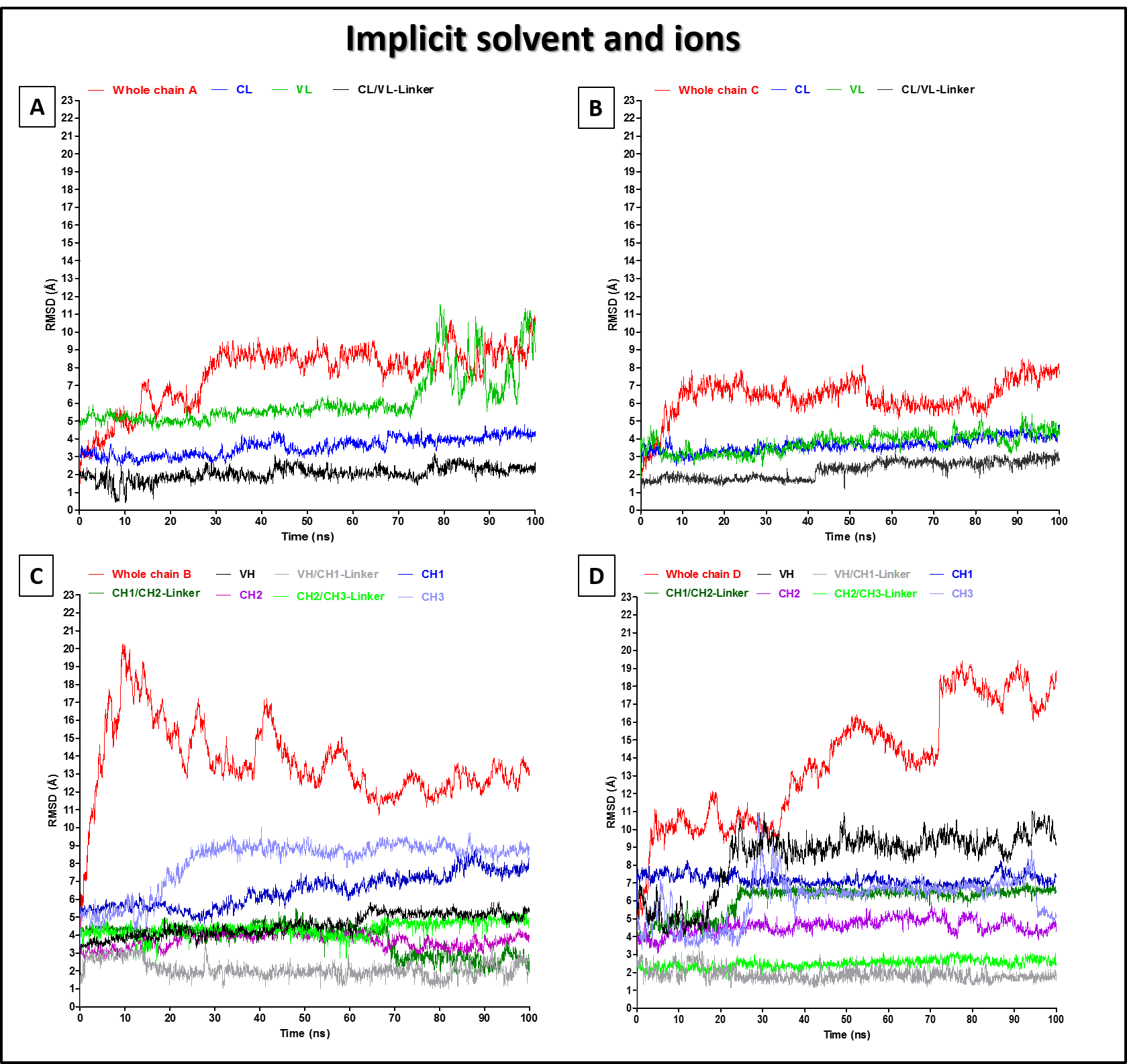


**Supplementary Figure S2: RMSD analysis of each chain in the implicit solvent and ions simulation**

The RMSD of various domains and linkers of the antibody four chains are illustrated in A) light chain A; B) light chain C; C) heavy chain B; D) heavy chain D. Each light chain is composed of variable (VL) and constant (CL) domains connected by a linker (CL/VL –linker). Whilst the heavy chain is composed of variable (VH) and three constant (CH1, CH2, and CH3) domains. Three linkers (VH/CH1-Linker, CH1/CH2-Linker, and CH2/CH3-Linker) connect the four domains of the heavy chains.


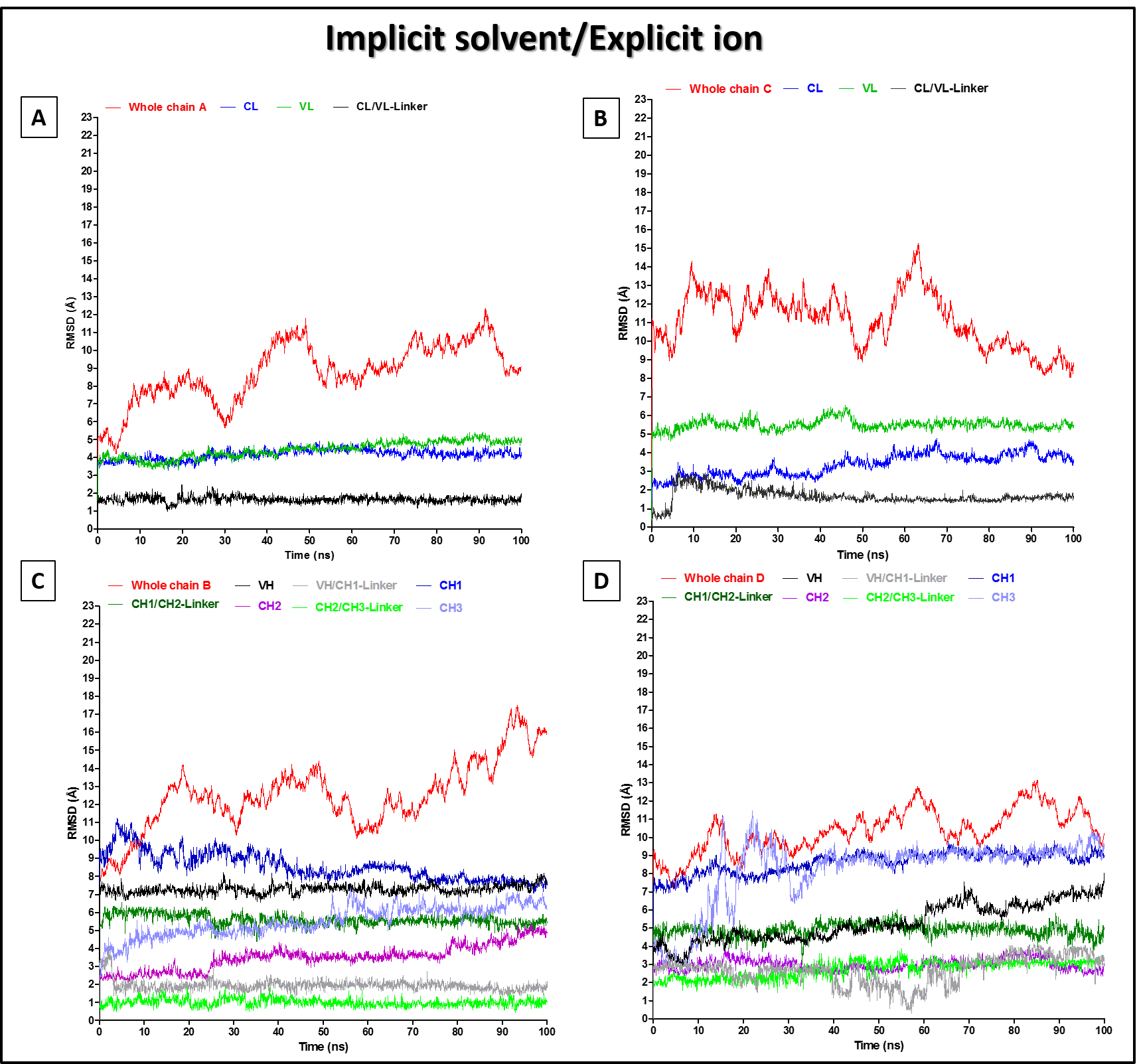


**Supplementary Figure S3: RMSD analysis of each chain in the implicit solvent/explicit ions simulation**

The RMSD of various domains and linkers of the antibody four chains are illustrated in A) light chain A; B) light chain C; C) heavy chain B; D) heavy chain D. Each light chain is composed of variable (VL) and constant (CL) domains connected by a linker (CL/VL –linker). Whilst the heavy chain is composed of variable (VH) and three constant (CH1, CH2, and CH3) domains. Three linkers (VH/CH1-Linker, CH1/CH2-Linker, and CH2/CH3-Linker) connect the four domains of the heavy chains.
